# Effects of dietary *Clostridium butyricum* on the growth performance, digestion, and intestinal health of spotted sea bass (*Lateolabrax maculatus*)

**DOI:** 10.3389/fimmu.2023.1181471

**Published:** 2023-07-14

**Authors:** Lumin Kong, Jianrong Ma, Sishun Zhou, Hao Lin, Zhongying Long, Huihui Qin, Longhui Liu, Yi Lin, Zhangfan Huang, Zhongbao Li

**Affiliations:** ^1^ Fisheries College, Jimei University, Xiamen, China; ^2^ Fisheries College of Jimei University, Fujian Provincial Key Laboratory of Marine Fishery Resources and Eco-environment, Xiamen, China

**Keywords:** *Clostridium butyricum*, spotted sea bass, growth performance, immunity, intestinal microbiota

## Abstract

*Clostridium butyricum* (CB) is known to promote growth, enhance immunity, promote digestion, and improve intestinal health. In this study, we investigated the effects of CB in the feed on growth performance, digestion, and intestinal health of juvenile spotted sea bass. To provide a theoretical basis for the development and application of CB in the feed of spotted sea bass, a total of 450 spotted sea bass with an initial body weight of (9.58 ± 0.05) g were randomly divided into six groups. Gradient levels with 0, 0.1%, 0.2%, 0.3%, 0.4%, and 0.5% of CB (1×10^9^ cfu/g) were supplemented into diets, designated as CC, CB1, CB2, CB3, CB4, and CB5, respectively. Each group was fed for 54 days. Our results suggest that dietary 0.2% and 0.3% of CB can significantly increase the weight gain (WG) and specific growth rate (SGR) of spotted sea bass. The addition of CB significantly increased intestinal amylase activity, intestinal villus length, intestinal villus width, and intestinal muscle thickness. Similarly, CB supplementation increased the expression of tumor necrosis factor-*α* (*TNF-α*) and interleukin-8 (*IL-8*). Sequence analysis of the bacterial 16S rDNA region showed that dietary CB altered the intestinal microbiota profile of juvenile spotted sea bass, increasing the dominant bacteria in the intestine and decreasing the harmful bacteria. Overall, dietary addition of CB can improve growth performance, enhance intestinal immunity, improve intestinal flora structure, and comprehensively improve the health of spotted sea bass.

## 1 Introduction

The spotted sea bass (*Lateolabrax maculatus*) belongs to the order Perciformes, the family Serranidae, and the genus *Lateolabrax* (1). The spotted sea bass lives in estuaries and is a wide temperature and salinity fish. Because of the culinary use of its meat, its rapid growth, and its strong adaptability, China began to expand its breeding scale as early as the 1970s, and now it is widely cultivated as an economic fish. With the rapid development of the aquaculture industry, problems such as environmental pollution and feed pollution have come one after another and directly lead to the aggravation of the intestinal disease problem of spotted sea bass and restrict the sustainable development of the spotted sea bass aquaculture industry, which is also a common problem in the aquaculture industry. Therefore, fish intestinal health and protection has attracted attention in the aquaculture industry. In recent years, there has been increasing interest in probiotics as a functional feed additive to regulate intestinal health. The term “probiotic” is derived from the Greek word for “life” and is defined as a “living microbial feed additive”, which improves the intestinal microbial balance of the host animal (2). It can improve intestinal flora structure, promote nutrient absorption and digestion, improve growth performance, enhance animal immunity, and prevent and control certain intestinal diseases (3–5). *Clostridium butyricum* (CB) has been widely used in livestock farming as a beneficial intestinal bacterium.

*Clostridium butyricum* (CB) is a strictly anaerobic Gram-positive bacillus (6). The bacterium is widely distributed and can live in the soil as well as in the intestines of any animal (7). The bacterium has good environmental adaptability and can produce spores to adapt to complex and changing environments. It is also resistant to stomach acid digestive juices, so it can maintain its role after entering the intestinal tract. The optimal growth temperature is 37°C, and at the same time, it does not inactivate at 100°C. In addition, CB can also be stored at room temperature. Previous studies have shown that CB inhibits the reproduction of harmful bacteria, promotes the reproduction of dominant bacteria, and regulates intestinal microecological balance (7, 8). It can also use carbohydrates in the large intestine to ferment short-chain fatty acids and repair intestinal mucosa (9, 10). In addition, CB can reduce the PH value of the intestinal environment, inhibit intestinal inflammatory response, and strengthen intestinal immune function and antioxidant capacity (11, 12). CB is resistant to most antibiotics (13). Current studies of CB on aquatic animals have been reported in yellow croaker (14), Tilapia (15), Hybrid grouper (16), etc. However, the application research of the basic feed used to make spotted sea bass has not been reported. It remains to be seen whether CB as a probiotic feed additive can improve the digestive ability and regulate the intestinal flora structure of spotted sea bass. Therefore, it is of great significance to explore the effect of CB as a feed additive on the intestinal health of perch, which can promote the green, healthy, and sustainable development of the aquaculture industry.

## 2 Materials and methods

### 2.1 Animal ethics

All procedures of this study were conducted on fish, and animal care was performed strictly following the Management Rule of Laboratory Animals (Chinese Order No. 676 of the State Council, revised 1 March 2017).

### 2.2 Diet formulation

Different levels of CB (1×10^9^cfu/g) were added to the base feed of spotted sea bass, 0(CC) for the control group, 0.1% (CB1), 0.2% (CB2), 0.3% (CB3), 0.4% (CB4), and 0.5% (CB5) and leveled with flour, and the additive CB was from Henan Jinbaihe Biotechnology Co., Ltd. The formulation and main components of the basal diet are shown in **Table 1**. Fish meal and soybean meal were first crushed and passed through 60 mesh sieves. With reference to the food preparation, each powder raw material and CB was mixed step by step, fish oil and soybean oil were added through the sieve, then approximately 30% water was added and mixed well. The pelletizer was used to make granular feed with a particle size of 2.5 mm, which was then put in the oven at 55°C and baked until the feed moisture reached approximately 10%; it was then removed, naturally dried, and stored in the refrigerator at -20°C.

**Table 1:** Basic feed composition and its components.

Ingredients	Contents (%)
Fish meal	49.0
soybean meal	23.5
Flour	15.0
Yeast powder	3.0
Fish oil	3.0
Soybean oil	2.0
Lecithin	1.0
Mineral premix ^(1)^	0.6
Vitamin premix ^(2)^	0.8
Choline	0.6
Ca(H_2_PO_4_)_2_	1.2
Multienzyme complex	0.3
Nutrient composition	
Crude protein	46.13
Crude fat	9.94
Total energy (kJ/Kg)^4^ Protein-energy ratio (mg prot/kJ)	16.56 27.85

(1) Mineral premix contains: MnSO_4_.4H_2_O 50 mg, MgSO_4_.H_2_O 4000 mg, CoCl_2_(1%) 100 mg, KI 100 mg, FeSO_4_.H_2_O 260 mg, CuSO_4_.5H_2_O 20 mg, ZnSO_4_.H_2_O 150 mg, Na_2_SeO_3_(1%) 50 mg.

(2) Vitamin premix contains: thiamine 25 mg, pyridoxine hydrochloride 20 mg, riboflavin 45 mg, VB_12_ 0.1 mg, VK_3_ 10 mg, inositol 800 mg, niacin 200 mg, pantothenic acid60 mg, biotin 1.2 mg, folic acid20 mg, VD_3_ 5 mg, VA acetate 32 mg, Ethoxy quinoline 150 mg, α-tocopherol 120 mg.

### 2.3 Experimental procedure

Laboratory animals were bought from Zhangpu Yanchang Jinxing Aquatic Co., Ltd. and kept in the aquaculture factory of the School of Aquatic Sciences, Jimei University, China. Before the start of the culture experiment, the fry was temporarily reared in a 1600 L cylindrical PVC barrel in the shed for two weeks and fed twice daily with a basal diet during the temporary rearing period. A total of 450 spotted sea bass of the same size (mean initial weight 9.58 ± 0.05) g were selected and randomly divided into six groups. Each group was repeated three times and assigned to 18 tanks for the experiment. In addition, each group was fed feeds with different CB contents, and the culture period was 54 days, during which they were fed twice daily at regular satiation (8:30,17:30). Water exchange of 30%-40% was performed daily. The concentration of ammonia nitrogen was less than 0.20 mg/L, the concentration of nitrite was less than 0.025 mg/L, and the concentration of dissolved oxygen was greater than 5.00 mg/L, with a pH of approximately 8.0.

### 2.4 Sample collection

At the end of the aquaculture, the spotted sea bass were fasted for 24 hours. After fasting, the fish were anesthetized with eugenol in each tank, and after anesthesia, the fish were retrieved, counted, and weighed to obtain the total weight. The intestines of six fish were randomly selected from each tank, rinsed with 0.86% ice saline to wash away the attached adipose tissue, put into liquid nitrogen for rapid freezing, and then transferred to a -80°C refrigerator for storage for digestive enzyme activity, intestinal flora, and related gene detection. The intestinal (approximately 1 cm) of three fish in each group were taken and stored in 4% paraformaldehyde for 24 h, transferred to 75% ethanol for hematoxylin and eosin staining, and used for morphological observation of the intestines.

#### 2.4.1 Growth performance

The fish growth performance index includes final weight (W_T_), weight gain (WG), specific growth rate (SGR), feed coefficient rate (FCR), and condition factor (CF), which are calculated as follows:

WG = (*W*_t_ − *W*_0_)/*W*_0_×100%

SGR = (ln *W*_t_ − ln *W*_0_)/*t*×100%

FCR= *F*/(*W*_t_ − *W*_0_)

CF = *W*_b_/*L* ^3^×100

Where *W*_t_ is final mean weight (g); *W*_0_ is initial mean mass (g); t is number of days (d); F is food intake (g); W_b_ is fish body mass (g); and *L* is fish body length (cm).

#### 2.4.2 Intestinal digestive enzyme activity

The samples of each group of spotted sea bass were dissected, and the intestine was isolated and added to pre-cooled saline (0.86%) in appropriate proportions (w/v) and homogenized by an adjustable high-speed centrifuge. The prepared homogenate was centrifuged at 4°C, 2500 r/min for 10 min. Then, the supernatant was collected for measurement. The amylase (AMS) activity was determined by α-amylase test kit, trypsin (TRS) by trypsin test kit, and lipase (LPS) by lipase kit, all from Nanjing Jiancheng Bio-Engineering Institute, China (Catalog No: AMS: C016-1-1, LPS: A054-1-1, TRS: A080-2).

#### 2.4.3 Histological observation of the intestine

Morphological changes in the intestinal tract of flowering bass were observed between groups using hematoxylin-eosin staining according to Shinde (17). Paraffin section preparation was first performed: tissue samples fixed in Bouin fixation were removed, washed three times with 70% alcohol, trimmed flat with scissors, and placed at the bottom of the tissue embedding box. The tissues were sequentially immersed in 70%, 85%, and 95% ethanol for 20 min each, then in 100% ethanol I and ethanol II for 10 min each. Then, 15 s of draining was required for each change of dehydrating agent. After dehydration, the tissues were immersed in alcohol-benzene solution for 10 min and then placed into xylene solution for 10 min to ensure the tissues were transparent. Immersion in xylene-paraffin wax equivalent mixture was done at 63°C. After the wax immersion was completed, the embedding cassette was stored in a 63°C oven to complete the embedding as soon as possible. After curing the paraffin wax, the slides were sliced by a microtome with a thickness of 4 μm. Structurally intact tissue spreads were selected, after which the labeled slides were baked in a 60°C oven. Next, H-E staining was performed: the slides were put into xylene I and II for 20 min each, anhydrous ethanol I and II for 5 min each, and 75% ethanol for 5 min and rinsed with tap water. Hematoxylin staining was performed, followed by eosin staining. After staining, the sections were sequentially transferred into anhydrous ethanol (I, II, and III) for 5 min each and sealed with neutral gum. Finally, the length and width of the intestinal villi and the thickness of the muscle layer were measured with the WT1000GM system.

#### 2.4.4 Intestinal immunity-related gene expression

Polymerase Chain Reaction was used to extract total RNA from the whole intestine of fish using a commercial kit (RC112, Nanjing Vazyme Biotech Co., Ltd, Nanjing, China) according to the manufacturer’s requirements and was electrophoresed on a 1.2% denaturing agarose gel to detect the quality. Then, extracted RNA was assessed by a Nano-800+ spectrophotometer (Shanghai Jiapeng Technology Co., LTD, China) to test the concentration. The cDNA was obtained by reverse transcription using a commercially available package (RC112-50, Nanjing Vazyme Biotech Co., Ltd, Nanjing, China). cDNA was quantified by fluorescence PCR using SYBR Green I chimeric fluorometric method, and the kit (Q711) was purchased from Nanjing Vazyme Biotech Co. β-actin was used as an internal reference gene, and the relative expression of individual genes between groups was calculated using the 2^-ΔΔCt^ method. The primers used were similar to those used in the previous study of the spotted sea bass (18) (**Table 2**).

**Table 2:** Primer sequences for RT-qPCR.

Gene	Forward	Tm, °C
β-actin	F: CCATCTATGAGGGCTACGC	60
	R: CGGCTGTGGTGGTGAAG	60
IL1β	F: CTGAACATCAAGGGCACAGA	60
	R: GTTGAAGGGGACAGACCTGA	60
IL8	F: GAGCTGATTCCTGCCAACTC	60
	R: CCGATCTGTTCAGGGTGTTC	60
TNF-α	F: GACTCCATAGGCAGCAAAGC	60
	R: AGAAAGTCTTGCCCTCGTCA	60
TGF-β	F: ACAGTGGGCAATGTAAGTGGT	60
	R: CTTGGTGCTGTGTGTAGAGGGA	60
IL10	F: ATTTCTACGAGGCAAACGACA	60
	R: TCCAGGCTGTGCGTATTTG	60

F means forward primer, while R means reverse primer.

#### 2.4.5 Intestinal microbial flora detection and analysis

The total bacterial genomic DNA of the intestinal samples was extracted by the TGuide S96 magnetic bead method, and the extracted genomic DNA was detected by 1.5% agarose gel electrophoresis. The upstream and downstream primers 338F (5’-ACTCCTACGGGAGGCAGCA-3’) and 806R (5’- GGACTACHVGGGTWTCTAAT-3’) were designed for the high mutation region V3-V4 of the 16S rRNA gene and PCR amplification was performed. PCR amplicons were quantitatively detected using 1.8% agarose gel and Image J software, and the quality of PCR amplicons was detected using the Qsep-400 method. Libraries were then created, and qualified libraries were sequenced using Illumina NovaSeq 6000. After pre-processing the sequencing data, bioinformatics analysis was performed. The products were sequenced on the Illumina NovaSeq by BMKCloud (www.biocloud.net) (Beijing, China).

#### 2.4.6 Statistical analysis

All relevant data obtained were statistically analyzed using SPSS22.0 software and analyzed by one-way analysis of variance (ANOVA), followed by Duncan’s multiple comparisons if the differences were significant, and all data were expressed as mean ± standard deviation (Mean ± SD), with *P*<0.05 selected as the significant level.

## 3 Results

### 3.1 Effects of CB on the growth performance of spotted sea bass

With the increase in CB content in the diet, the growth performance of spotted sea bass showed a trend of increasing and then decreasing (**Table 3**). When added at 0.2% - 0.3%, the WG and SGR were significantly higher than the control group (p<0.05). There was no significant difference between FCR and CF (*P*>0.05).

**Table 3:** Effects of CB on growth performance.

GROUP	W_T_ (g)	WG (%)	SGR (%/day)	FCR	CF (g/cm^3^)
CC: control	60.89 ± 0.91^a^	536.2 ± 10.07^a^	3.43 ± 0.03^a^	1.14 ± 0.01^a^	1.08 ± 0.06^a^
CB1: 0.1% CB	62.51 ± 2.3^ab^	551.38 ± 21.72^ab^	3.47 ± 0.06^ab^	1.09 ± 0.02^a^	1.03 ± 0.08^a^
CB2 0.2% CB	65.31 ± 3.6^b^	581.8 ± 37.57^b^	3.55 ± 0.1^b^	1.1 ± 0.07^a^	1.06 ± 0.06^a^
CB3 0.3% CB	65.5 ± 1.8^b^	583.97 ± 19.26^b^	3.56 ± 0.05^b^	1.08 ± 0.03^a^	0.99 ± 0.02^a^
CB4 0.4% CB	62.5 ± 0.96^ab^	551.81 ± 8.73^ab^	3.47 ± 0.02^ab^	1.05 ± 0.07^a^	1.07 ± 0.02^a^
CB5 0.5% CB	63.11 ± 0.55^ab^	557.94 ± 3.97^ab^	3.49 ± 0.01^ab^	1.07 ± 0.04^a^	1.05 ± 0.07^a^

Different superscript letters in a column indicate significant difference (P<0.05).

### 3.2 Effect of CB on the activity of intestinal digestive enzymes and the intestinal tissues of spotted sea bass

The effect of CB supplementation in the diet on the activity of intestinal digestive enzymes (**Table 4**). One-way ANOVA showed that AMS activity increased significantly with increased CB addition (*P*<0.05), with the most significant addition at CB5. TRS and LPS showed a trend of first increasing and then decreasing, but not significantly. (*P*>0.05). As shown in **Table 5**, intestinal wall thickness, intestinal villus length, and intestinal villus width increased significantly due to increased CB dose groups (*P*<0.05), with the most significant addition at CB5.

**Table 4:** Effect of CB on the activity of intestinal digestive enzymes.

GROUP	AMS (U/mg prot)	LPS (U/g prot)	TRS (U/mg prot)
CC: control	0.21 ± 0.02^a^	2.72 ± 0.66^a^	222.16 ± 31.35^a^
CB1: 0.1% CB	0.35 ± 0.09^b^	3.15 ± 0.8^a^	334.52 ± 97.8^a^
CB2 0.2% CB	0.34 ± 0.03^b^	2.37 ± 0.42^a^	238.58 ± 19.32^a^
CB3 0.3% CB	0.41 ± 0.07^bc^	2.14 ± 0.23^a^	280.06 ± 51.09^a^
CB4 0.4% CB	0.46 ± 0.06^cd^	2.43 ± 0.56^a^	236.89 ± 75.18^a^
CB5 0.5% CB	0.52 ± 0.05^d^	2.29 ± 0.22^a^	226.6 ± 64.99^a^

Different superscript letters in a column indicate significant difference (P<0.05).

**Table 5:** Effect of CB on the intestinal tissues.

GROUP	Intestinal muscle thickness (um)	Intestinal villus length (um)		Intestinal villi width (um)	
CC: control	182.62 ± 29.13^a^	341.94 ± 62.33^a^		58.25 ± 14.54^a^	
CB1: 0.1% CB	214.06 ± 15.08^ab^	514.88 ± 112.6^b^		74.48 ± 7.23^b^	
CB2 0.2% CB	223.99 ± 42.05^bc^	563.85 ± 57.41^b^		69.73 ± 13.98^ab^	
CB3 0.3% CB	248.61 ± 25.31^bc^	620.89 ± 191.26^bc^		66.28 ± 12.92^ab^	
CB4 0.4% CB	235.12 ± 46.31^bc^	679.48 ± 45.98^c^		73.21 ± 5.09^b^	
CB5 0.5% CB	255.02 ± 42.22^c^	679.99 ± 88.96^c^		77.74 ± 14.62^b^	

Different superscript letters in a column indicate significant difference (P<0.05).

### 3.3 Effects of CB on the expression of intestinal-related factors in spotted sea bass

**Figure 1:**
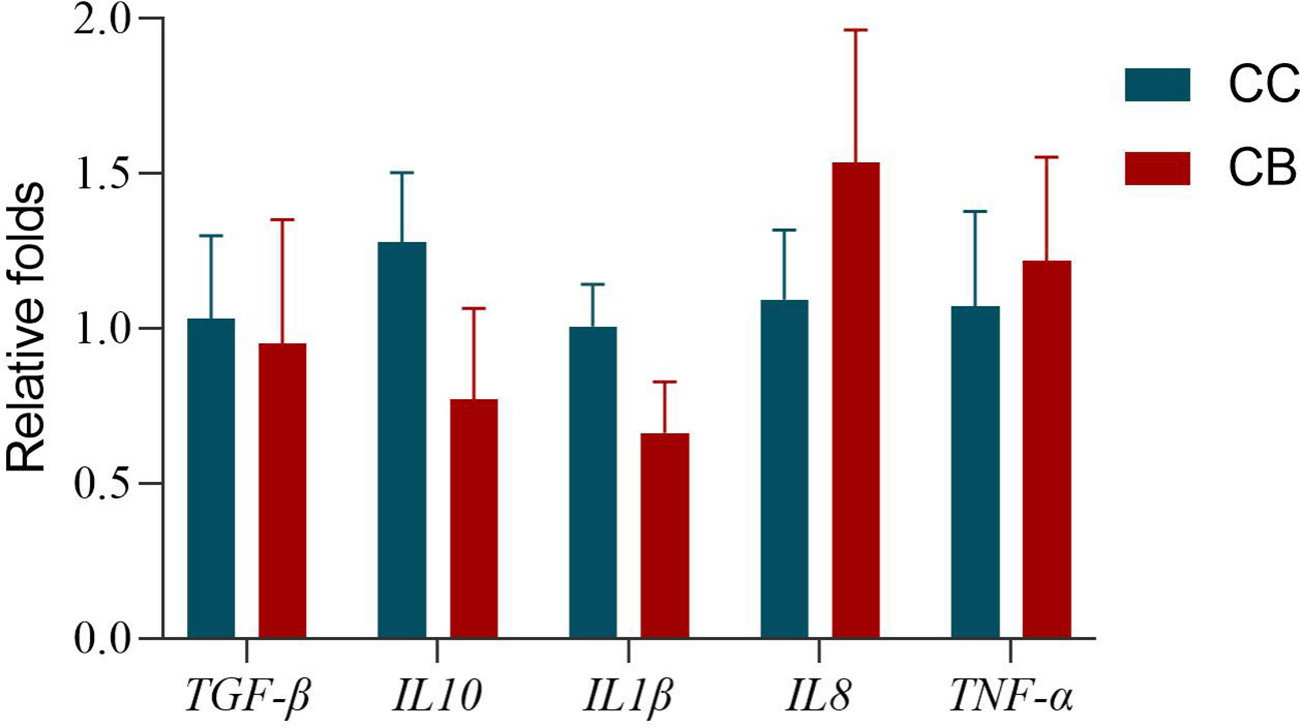
Effects of early life intervention using *Clostridium butyricum* on *TGF-β*, *IL10*, *IL1β*, *IL8*, and *TNF-α* mRNA expression in the whole intestine of spotted sea bass. Values are means (n = 3), with their standard errors represented by vertical bars. Bars bearing the same letters were not significantly different (P > 0.05).

The expression of intestinal-related factors in CB added to the diet is shown in **Figure 1**. The expression of pro-inflammatory cytokine *IL1β* was lower than that of the control group, while the expression of *TNF-α* and *IL8* genes was higher than that of the control group: 0.65 times, 1.4 times, and 1.13 times, respectively. The expression of anti-inflammatory cytokines *TGF-β* and *IL10* was lower than that of the control group: 0.92 times and 0.6 times, respectively.

### 3.4 Effect of CB on the intestinal microbial structure

**Figure 2:**
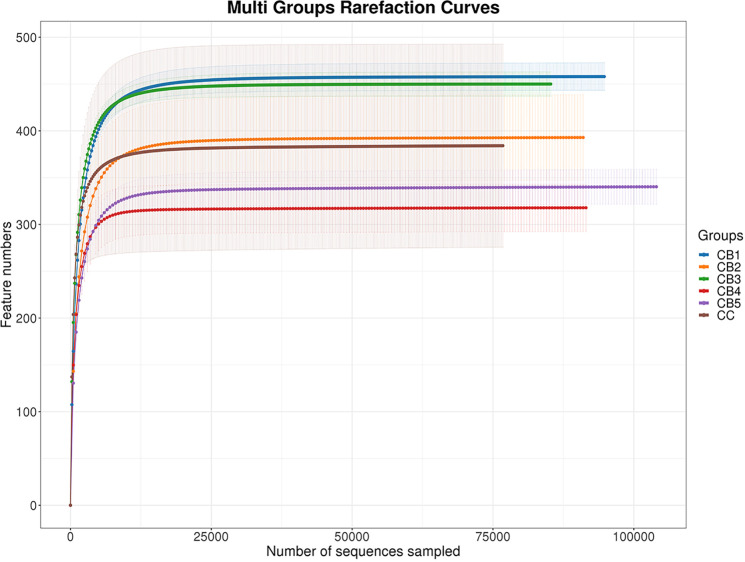
The number of detected species will be growing, with more sequencing data included. Once the counts of species reach saturation, there will not be more species detected with more sequencing data sampled.

Once assembled, quality screened, and trimmed, a total of 2879006 high-quality valid reads were obtained, ranging from 159217 to 161017, resulting in the identification of 1082 OTUs with 97% identity from 12 samples (data not shown). The OTUs were assigned to 446 species, 414 genera, 242 families, 140 orders, 51 classes, and 26 phyla (data not shown). For all samples, the sparse curve of OTU number tends toward saturation, indicating that all samples were fully sequenced (**Figure 2**).

**Figure 3:**
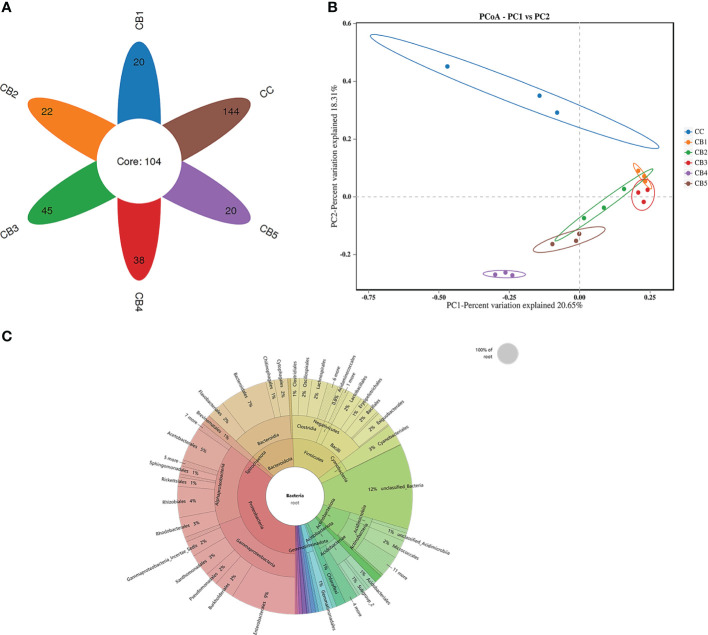
Effects of early life intervention using *Clostridium butyricum* on the intestinal microbial structure of juvenile spotted sea bass **(A)** Venn diagram; **(B)** Principal coordinates analysis (PCoA); **(C)** KRONA Species Notes.

Venn (**Figure 3A**) showed that all groups shared 104 OTUs, while the number of unique OTUs in CC, CB1, CB2, CB3, CB4, and CB5 was 144, 20, 22, 45, 38, and 20, respectively. The results of the diversity index showed that Alpha indices showed a tendency to increase in ACE and Chao1 indices compared to the control group but were not significant (*P*>0.05), and the addition of excess CB resulted in a decrease in their species number. Simpson and Shannon indices significantly decreased, except for the CB3 group (*P*<0.05) (**Table 6**). To analyze the degree of similarity of microbial communities, principal component analysis was performed to determine β-diversity. Principal coordinate analysis (PCoA) was used to explore the similarity of samples after adding different doses of CB (**Figure 3B**). The results of the sample species annotation using KRONA v2.6 revealed that the gut microorganisms of the flowering spotted sea bass Proteobacteria accounted for 34%, Firmicutes for 15%, unclassified bacteria for 13%, Bacteroidota for 10%, Actinobacteriota for 9%, Acidobacteriota for 6%, Cyanobacteriales for 4%, Gemmatimonadota and Chloroflexi for 1%, etc. (**Figure 3C**).

**Table 6:** Alpha diversity index statistics.

GROUP	ACE	Chao1	Simpson	Shannon
CC: control	386.14 ± 105.55^ab^	385.67 ± 106.65^ab^	0.98 ± 0.01^bc^	7.48 ± 0.5^c^
CB1: 0.1% CB	458.19 ± 14.84^b^	458.33 ± 15.18^b^	0.96 ± 0.02^bc^	6.67 ± 0.38^b^
CB2 0.2% CB	393.49 ± 45.83^ab^	394 ± 47.13^ab^	0.94 ± 0.02^b^	6.16 ± 0.4^ab^
CB3 0.3% CB	450.05 ± 13.04^b^	450 ± 13.00^b^	0.99 ± 0.02^c^	7.56 ± 0.07^c^
CB4 0.4% CB	325.51 ± 15.33^a^	319 ± 23.52^a^	0.95 ± 0.03^bc^	6.42 ± 0.6^b^
CB5 0.5% CB	346.81 ± 20.89^a^	342.17 ± 19.94^a^	0.89 ± 0.04^a^	5.56 ± 0.42^a^

Different superscript letters in a column indicate significant difference (P<0.05).

**Figure 4:**
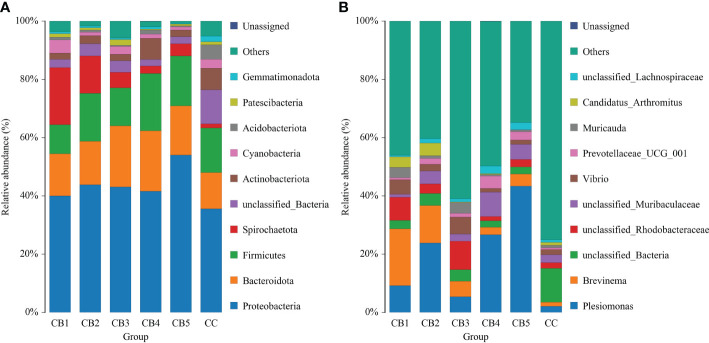
Taxonomy classification of reads at phylum **(A)** and genus **(B)** taxonomic levels (n = 3/group). Note: X-axis: Sample IDs; Y-axis: Relative abundance.

**Figure 5:**
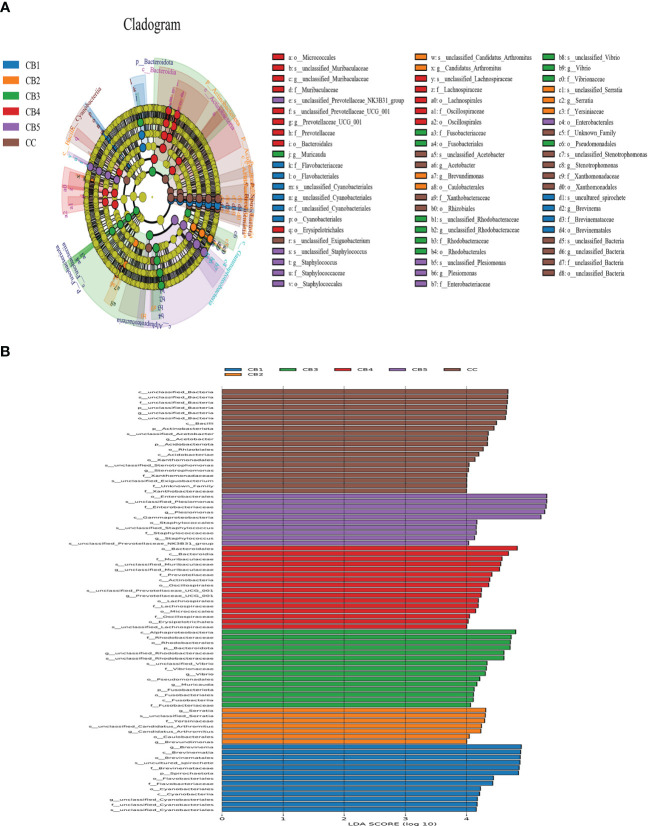
LEfSe analysis identified the most differentially abundant taxons between the Control and Clostridium butyricum supplementation groups (n = 3/group). **(A)** Evolutionary branching diagram for LEfSe analysis. Differences are indicated by the color of the most abundant taxon (yellow indicates no significant difference). **(B)** Histogram of linear discriminant analysis (LDA) scores for differentially abundant taxon. Cladogram was calculated by LEfSe.

At the phylum level, the dominant bacteria in the intestinal of the CC and CB were Proteobacteria, Bacteroidota, and Firmicutes. Compared with CC (0%) of the control group, Proteobacteria, Bacteroidota, and Firmicutes of the experimental group were increased (**Figure 4A**). Spirochaetota increased compared with the CC group. At the genus level, *Plesiomonas* and *Brevinema* were the dominant bacteria. Compared to the CC group, the unclassified Bacteria reduced. In addition, CB increased the proportion of the advantage bacterium (**Figure 4B**); Lefse analysis was performed to compare the difference in intestinal microbial community composition between the control group and the CB. The results show that the addition of CB significantly increased the Fusobacteriota phylum, the Gammaproteobacteria class, the Bacteroidota order, the Fusobacteriaceae family, and the *Candidatus Arthromitus genus* and significantly reduced the unclassified_Bacteria, Actinobacteriota, and Acidobacteriota phyla and the *Stenotrophomonas* genus (*P*<0.05) (**Figures 5A, B** **)**.

## 4 Discussion

CB is a probiotic bacterium that colonizes the intestinal tract of humans and animals. In livestock and aquaculture, CB is widely used to promote animal growth, facilitate digestion, and improve meat quality (19, 20). In the present study, we show that the addition of CB (0.2%-0.3%, 1 × 10^9^ cfu/g) to the diet of spotted sea bass results in significant weight gain, indicating that appropriate concentrations of CB can significantly promote the growth of spotted sea bass. This result is consistent with previous studies of CB on *Litopenaeus vannamei* and *Tilapia* (21, 22). The growth improvement effect of probiotics on spotted sea bass has been well established, such as *Bacillus licheniformis* and *Bacillus subtilis* with Asian sea bass (23). *Lactococcus petaurid* and Bacillus *siamensis* with *Lateolabrax japonicus* (24). The positive effect of CB on the growth performance of animals may be related to its production of short-chain fatty acids and digestive enzymes during metabolism, which promote the body’s ability to absorb and utilize nutrients (25). Notably, the trend of increasing and then decreasing WG and SGR is consistent with the findings of Sumon et al. (26) and Poolsawat et al. (27), which may be due to the imbalance of intestinal flora caused by excess CB, resulting in reduced growth performance. In the following paragraphs, we will further discuss their effects on digestive enzymes, intestinal morphology, inflammatory immunity, and microbiota.

As an important digestive organ of fish, the activity of its digestive enzymes is closely related to the growth and development of fish and its intestinal health, and the activity of digestive enzymes can directly reflect the degree of digestion and absorption of nutrients in the intestine of fish (28, 29). The study of digestive enzymes in aquatic animals mainly includes amylase, lipase, and trypsin. Similar to the present study, Luo et al. (30) found that CB significantly increased the amylase activity of grass turtles. *Bacillus licheniformis* and *Bacillus subtilis* increased the activity of amylase, lipase, and trypsin in Asian sea bass. In this study, amylase activity increased significantly with the increase in CB content, but there was no difference between its trypsin and amylase activities. This may be due to the ability of CB to ferment carbohydrates to produce large amounts of butyric acid and pyruvic acid, thus increasing amylase activity, while other probiotics mainly promote intestinal digestion and absorption by secreting exogenous enzymes (31).

The structural integrity of the intestine affects the absorption of nutrients by the organism (32). It is now generally accepted that the morphological structure of the intestine and the growth performance of the animal are related to the activity of digestive enzymes (33). The length and width of the villi in the intestine are important morphological parameters of intestinal function. In this study, adding CB significantly increased the intestinal villus length, intestinal villus width, and intestinal wall thickness of the spotted sea bass. Previous studies have shown that the addition of CB to the diet of Litopenaeus vannamei significantly increases the height of the intestinal villi and the thickness of the intestinal muscle layer. After adding CB to the feed of Yellow Catfish, the length and width of its villi increased significantly, and the growth performance could also be improved significantly (34). Thus, CB promotes the digestion and absorption of nutrients in the intestine by improving the intestinal structure. This suggests that CB can improve intestinal health by improving intestinal tissue morphology.

Many studies have demonstrated the application of CB to enhance intestinal immunity (35). The intestinal immune response was associated with cytokine-mediated inflammatory response (36). Tumor necrosis factor α (*TNF-α*) and interleukin 1β (*IL1β*) are typically pro-inflammatory cytokines, and up-regulation of their gene expression is closely associated with enteritis (37). In contrast, interleukin 10 (*IL10*) is an anti-inflammatory cytokine (38). In this study, the mRNA expression of *IL1β* and *IL10* genes was downregulated in the intestinal of the CB group, indicating that the CB group of spotted sea bass showed a significant decrease in inflammatory response. Sui et al. (39) found that CB significantly increased the expression of *IL8* in interstitial cells of Cajal. Gao et al. (40) found significantly up-regulated *TNF-α* and *IL8* in HT-29 cells. Sodium butyrate up-regulated *TNF-α* in the intestinal cells of carp (41). This means that Cytokine regulation by CB may be dependent on butyric acid metabolites. In this experiment, the addition of CB to the diet up-regulated the gene expression of *TNF-α* and *IL8* in the intestinal tract of spotted sea bass. This experimental result suggests that the addition of appropriate levels of CB to the diet can stimulate the intestinal immune response and increase intestinal immunity.

The internal environment of the animal body is very complex, and the intestinal flora is an important element of this. The intestinal flora of fish is a complex ecosystem that is affected by aquaculture, the environment, food, and various stages of growth and development. A better understanding of the role of intestinal flora in fish health is a key factor in the sustainable development of aquaculture. The regulation of gut microbes by probiotics has long been established. Previous results have suggested that beneficial bacteria can regulate the structure and composition of animal intestinal flora (35, 42, 43). In the present study, ACE and Chao1 indices in the intestine of juvenile fish increased and then decreased compared to the control group, probably due to the influence of different concentrations of CB on the intestinal microbial richness of the spotted sea bass. CB supplementation significantly increased beneficial bacteria, such as the Fusobacteriota class, the Bacteroidota order, and Emilia Fusobacteriaceae, while significantly decreasing harmful bacteria such as unclassified_Bacteria and the Actinobacteriota phylum. These findings suggest that different concentrations of CB have different effects on the number of intestinal species, which is consistent with the results regarding growth performance. In contrast, the intestinal microbial diversity of spotted sea bass was reduced, which may be due to the decrease in its biodiversity because of the decrease in harmful bacteria species caused by the increase in CB. Previous results showed that Yin et al. (14) found that CB supplementation at 0.10 and 0.20% levels significantly reduced the relative abundance of the Actinobacteria phylum, suggesting that CB at 0.10 and 0.20% may have a more profound effect on the gut community of larvae than CB at 0.40%. Poolsawat et al. (27) fed a CB-supplemented diet to tilapia for 8 weeks. The relative abundance of planarians, proteobacteria, and chlorophyll was significantly higher in probiotic-fed tilapia than in controls. *Candidatus Arthromitus* has the unique ability to modulate the host immune response. In this study, the abundance of *Candidatus Arthromitus* was significantly increased (44). In conclusion, *Clostridium butyricum* maintains intestinal health, mainly through mechanisms such as promoting the balance of intestinal microbial communities, regulating the immune system, and inhibiting the growth of harmful bacteria.

## 5 Conclusion

The present study shows that the addition of 0.2% - 0.3% CB (1×10^9^ cfu/g) to the feeds promotes the growth performance of juvenile spotted sea bass. In addition, CB improves growth performance by promoting intestinal development, increasing digestive enzyme activity, regulating the dominant intestinal bacteria, and improving intestinal health. These findings provide a theoretical basis for the application of CB as a feed additive to promote the growth and intestinal health of spotted sea bass in aquaculture.

## Data availability statement

The datasets presented in this study can be found in online repositories. The names of the repository/repositories and accession number(s) can be found below: https://figshare.com/, https://doi.org/10.6084/m9.figshare.22344946.v1.

## Ethics statement

The animal study was reviewed and approved by Animal Ethics Committee of Jimei University.

## Author contributions

LK and ZBL carried out the animal experiments and data analysis and drafted the manuscript. LK, JM, SZ, and HL participated in the animal trial. ZH, YL, LL, ZYL, and HQ were responsible for sample collection. LK analyzed the data, wrote the paper, and prepared figures and tables. ZBL reviewed the drafts of the paper. All authors contributed to the article and approved the submitted version.
